# Impact of an Innovative Equipment to Monitor and Control Salt Usage during Cooking at Home on Salt Intake and Blood Pressure—Randomized Controlled Trial iMC SALT

**DOI:** 10.3390/nu14010008

**Published:** 2021-12-21

**Authors:** Tânia Silva-Santos, Pedro Moreira, Olívia Pinho, Patrícia Padrão, Sandra Abreu, Sílvia Esteves, Luís Oliveira, Pedro Norton, Micaela Rodrigues, Altin Ndrio, Carla Gonçalves

**Affiliations:** 1 Faculty of Nutrition and Food Sciences, University of Porto, 4150-180 Porto, Portugal; pedromoreira@fcna.up.pt (P.M.); oliviapinho@fcna.up.pt (O.P.); patriciapadrao@fcna.up.pt (P.P.); micaela.rodrigues97@hotmail.com (M.R.); carlagoncalves.pt@gmail.com (C.G.); 2 EPI Unit—Institute of Public Health, University of Porto, 4200-450 Porto, Portugal; pnorton@med.up.pt; 3 CIAFEL—Research Centre in Physical Activity, Health and Leisure, Faculty of Sport, University of Porto, 4200-450 Porto, Portugal; sandramrabreu@gmail.com; 4 Laboratório Para a Investigação Integrativa e Translacional em Saúde Populacional, 4050-600 Porto, Portugal; 5 LAQV-REQUIMTE—Laboratory of Bromatology and Hydrology, Faculty of Pharmacy, University of Porto, 5000-801 Porto, Portugal; 6 Faculty of Psychology, Education and Sports, Lusófona University of Porto, 4000-098 Porto, Portugal; 7 INEGI—Institute of Science and Innovation in Mechanical and Industrial Engineering, University of Porto, 4200-465 Porto, Portugal; sesteves@inegi.up.pt (S.E.); loliveira@inegi.up.pt (L.O.); 8 Occupational Health Service, Centro Hospitalar Universitário de São João, EPE, 4200-319 Porto, Portugal; 9 Clinical Pathology Service, Centro Hospitalar Universitário de São João, EPE, 4200-319 Porto, Portugal; altin.ndrio@chsj.min-saude.pt; 10 CITAB—Centre for the Research and Technology of Agro-Environmental and Biological Sciences, University of Trás-os-Montes and Alto Douro, 5000-801 Vila Real, Portugal

**Keywords:** dietary sodium, sodium restricted, hypertension, RCT, cardiovascular disease

## Abstract

(1) Background: Excessive salt consumption is associated with an increased risk of hypertension and cardiovascular disease, and it is essential to reduce it to the level recommended by the World Health Organization (<5 g/day). The main objective of this study is to verify the impact of an intervention, which used the Salt Control H equipment to reducing salt consumption; (2) Methods: The study was an 8-week randomized control trial with 114 workers from a public university. The intervention group (*n* = 57) used the equipment to monitor and control the use of salt during cooking (Salt Control H) at home for 8 weeks. The primary outcome was 24 h urinary sodium excretion as a proxy of salt intake. Secondary outcomes included changes in 24 h urinary potassium excretion, sodium to potassium ratio (Na:K), and blood pressure. (3) Results: There was a decrease in sodium intake after the intervention but with no statistical significance. When analyzing the results by sex and hypertension status, there was a reduction in sodium (−1009 (−1876 to −142), *p* = 0.025) and in Na:K ratio (−0.9 (−1.5 to −0.3), *p* = 0.007) in hypertensive men in the intervention group. (4) Conclusions: Interventions with dosage equipment can be valid approaches in individual salt reduction strategies, especially in hypertensive men.

## 1. Introduction

Excessive salt intake is associated with an increased risk of hypertension and cardiovascular disease [1]. It is estimated that 3 million of deaths worldwide are associated with high salt intake [2], making it essential to reduce salt intake.

There is evidence that reducing salt intake reduces blood pressure [1,3], in addition to bringing other health benefits in terms of cardiovascular disease and other chronic conditions [4].

In Portugal, in 2014, the main risk factors that contributed to the total disability-adjusted life years were inadequate eating habits, with low fruit intake and salty food intake being the main responsible factors [5].

The average intake of salt in Portuguese adults (10.7 g/day) is higher than recommended by the World Health Organization (5 g/day) [6,7] and similar to the global average for salt (10.06 g/day) [8]. The main source in Portugal is the salt added in the preparation and cooking, corresponding to 25 to 50% of the consumed salt [9,10]. Therefore, innovative interventions to reduce salt intake at the household level should be a priority in the country.

The reduction of population salt consumption was identified by the World Health Organization as one of the five major priority interventions to prevent non-communicable diseases based on criteria such as health effects, cost-effectiveness, low implementation costs, and political and financial viability. The goal is to reduce world salt consumption to less than 5 g per person per day by 2025 [11]. However, despite efforts to combat high dietary salt intake [9,12], progress remains slow.

Where the main source of salt intake is through salt added at the table or during cooking, education and communication strategies are important to influence consumer behavior to reduce salt intake [7]. A quick and easy-to-use instrument to monitor and control the salt added to foods during cooking may be the solution as consumers are not aware of the amount of salt consumed [7].

Thus, the main objective of this study is to verify the impact of an intervention, which is the use of the Salt Control H equipment in reducing salt consumption.

## 2. Materials and Methods

The trial design was registered at clinicaltrials.gov (accessed on 20 November 2021) (NCT03974477), and a detailed description of the methods of the iMCSalt study has been published previously [13].

### 2.1. Study Design and Participants

The study was an 8-week randomized controlled trial. In total, 139 participants were recruited from routine occupational health appointments of the University of Porto (white-collar and blue-collar workers). Recruitment was carried out by the doctor responsible for the appointment and started in June 2019 and ended in January 2021. The last participant evaluations ended in September 2021. The participants were evaluated four times: first at baseline, then two times during the intervention (week 4 and week 8, at end of intervention) and 6 months after the end of the intervention.

The following inclusion criteria were used by the doctor responsible for the recruitment: adult (>18 years), frequent home-cooked meals (more than 4 days a week and at least 3 Sundays per month), and reports motivation to control dietary salt consumption. The exclusion criteria were pregnancy, with hypotension, active infection that impacts renal function, kidney disease, urinary incontinence, acute coronary syndrome, severe liver disease or heart failure, member of the faculty that promotes the study (i.e., Faculty of Nutrition and Food Sciences), and not using salt for cooking.

The sample size calculated a priori was 260 participants [13], which was calculated to provide a significance level of 0.05 two-tailed and more than 80% statistical power, to assume a difference in 24 h mean urinary sodium excretion equal to or greater than 27 mmol/day between the intervention group and the control group, with a SD of 70 mmol/day [14].

### 2.2. Randomization

Included participants were randomly allocated to the control or intervention group (ratio 1:1), using computerized random numbers. Randomization of participants was stratified according to sex (ratio 1:1) and diagnosis of arterial hypertension (ratio 0.4:0.6) [6].

Randomization was performed after recruitment by a researcher independent of the recruitment and intervention process. The allocation sequence was concealed until the baseline assessment; participants and researchers discovered which group the participant belonged to at the same time by opening opaque envelopes identified with the participant’s identifier number.

### 2.3. Intervention

The intervention consisted of using the Salt Control H equipment by the participants at home to control salt quantity for cooking all meals during the intervention period [13].

**Figure 1:**
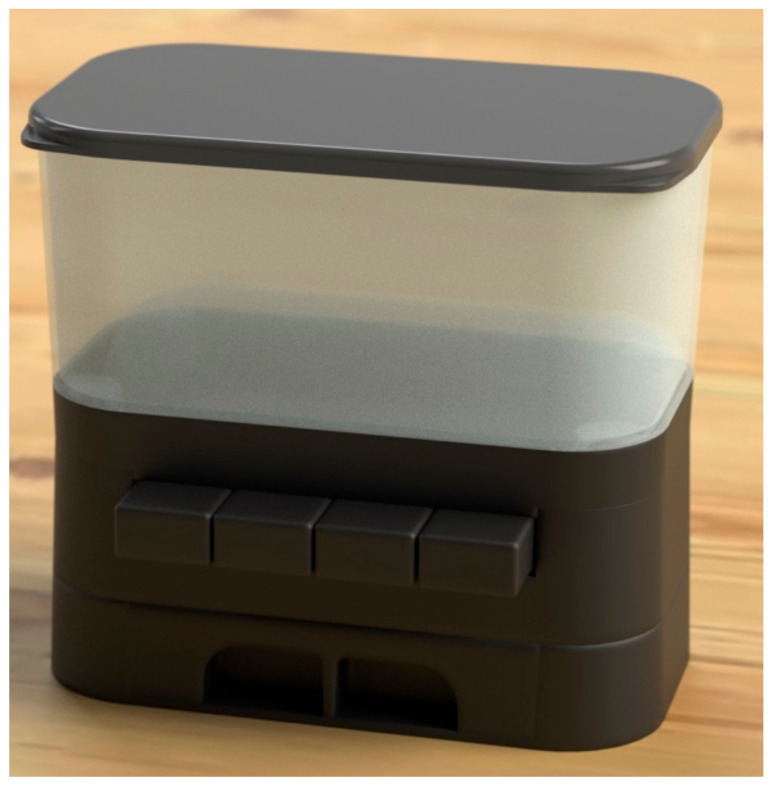
Salt Control H prototype.

Salt Control H is a dispenser that offers doses of salt according to the number of persons and their age (child or adult) of the consumers. The equipment dispenses 0.8 g or 0.2 g of salt for adult and child meals, respectively. It dispenses 0.2 g or 0.1 g of salt for every 250 mL of adult soup or children’s soup, respectively (Figure 1). Participants in the intervention group received a standardized presentation session that explained, through a video tutorial, how the salt control equipment works to ensure adequate salt content during food preparation and cooking. The researcher also provided some cooking strategies to prepare meals with adequate salt content. The prototype is only available for testing and not for commercial distribution. The Salt Control H equipment has been patented (INPI, No. 20191000033265) [15].

### 2.4. Control Group

We made the Portuguese food guide [16] available to all participants, whether in the intervention group or the control group. This guide consists of seven food groups with serving recommendations for each group.

### 2.5. Outcomes

#### 2.5.1. Primary and Secondary Outcomes

The primary outcome of this study was the difference in 24 h urinary sodium between the intervention group and the control group from the baseline to the end of the intervention (week 8). The secondary outcomes were the differences in urinary potassium excretion, Na:K ratio, and systolic and diastolic blood pressure between the two groups from the baseline to the end of the intervention (week 8).

These outcomes were evaluated four times: at baseline, during the intervention (week 4), at the end of the intervention (week 8), and six months after the end of the intervention (follow up).

The 24 h urine collection as a proxy of salt intake was performed any day of the week (preferably Sunday, but not Friday and Saturday due to laboratory availability). All participants for each evaluation were given one standard sterilized and coded container for urine collection and an illustrative leaflet with the collection procedure.

The following parameters were analyzed: volume, sodium (by indirect potentiometry), potassium (by indirect potentiometry), and creatinine (by photometry). The validity of 24 h urine collection was assessed by the relationship between urinary creatinine (mg/day) and body weight (kg). Samples were excluded when creatinine (mg/day/kg) was <10.8 and >25.2 in women and <14.4 and >33.6 in men [17]. Six urines were excluded in the intervention group and 10 urines were excluded in the control group.

The conversion of mmol to mg was performed by multiplying sodium (mmol) and potassium (mmol) respectively by 23 or 39 (mmol = mg/atomic weight). The Na:K ratio was obtained by dividing sodium (mmol) by potassium (mmol).

Blood pressure was measured using a portable sphygmomanometer (Edan M3A, Edan Instruments, Shenzhen, China) and was performed with the participants seated and after a 5 min rest. Blood pressure measurements were taken on both arms to determine possible arm-related measurement differences. A new measurement was taken on the arm that gave the highest value, and an average of the values of the two measurements was taken. If the difference between the first two assessments in the same arm was greater than 10 mm Hg, another third assessment was performed, and the mean was taken from the values of the last two assessments.

#### 2.5.2. Sample Characterization and Adjustment Variables

The sociodemographic questionnaire was based on the WHO STEPS questionnaire [18], and the skin phenotype was characterized according to the Fitzpatrick classification, which was classified as redhead with freckles, blonde, brunette, Latino, Arab, Asian, or black [19].

The level of physical activity was assessed using the International Physical Activity Questionnaire-Short Form, which was validated and adapted for the Portuguese population [20].

Height was measured by a stadiometer (SEA 213 portable stadiometer, Hamburg, Germany) measured according to international standard procedures [21]. The weight was measured with the Tanita MC180MA body composition analyzer (Tanita, IL, USA), after introducing the height calculated the body mass index (BMI). Measurements were taken with participants wearing light clothing and no shoes.

Food intake was assessed using a 24 h dietary recall. Participants were asked to recall all foods and beverages consumed the day before (time of urine collection) and estimated the portion size with the aid of a picture book. Energy and nutritional intake were estimated using the Food Processor Plus nutritional analysis software (version 11.9, ESHA Research, Salem, OR, USA).

The participants’ diagnosis of hypertension was made by the doctor responsible for the recruitment visit.

Participants who dropped out or did not agree to participate in the study filled out a questionnaire on simple sociodemographic questions.

### 2.6. Blinding

Participants and researchers were not blinded because the Salt Control H equipment cannot be masked. The 24 h urine analysis was performed by laboratory technicians who were unaware of the origin of the samples for analysis, and the researcher responsible for the randomization was not involved in the recruitment nor had any contact with the participants during data collection.

### 2.7. Statistical Methods

Statistical analysis was performed using the Statistical Package for the Social Sciences (SPSS Version 27). All statistical analyses and confidence intervals were two-sided, with *p* < 0.05 regarded as significant.

The normality of the variables was assessed using the Kolmogorov–Smirnov statistical test. Descriptive statistics were performed according to the characteristics of the variables, and the baseline differences between the intervention group and the control group were analyzed by the independent t-test (variables with normal distribution) or the Mann–Whitney U-test (variables with non-normal distribution) for continuous variables and χ2 test for categorical variables. Variables were presented as mean ± standard deviation (variables with normal distribution) or mean [P25; P75] (variables with non-normal distribution).

The difference in 24 h urinary sodium and potassium excretion and blood pressure was made using linear mixed models with an intention-to-treat approach with analyzed participants according to initial randomization [22]. Multiple imputations were performed to obtain a data set with no missing values. Models with health assessments (baseline, week 4, week 8 and follow up) and treatment group (intervention vs. control group) as fixed effect and person as random effect were performed to test the change from baseline to the 8-week follow-up in 24 h urinary sodium and potassium excretion and blood pressure. Autoregressive structure with heterogenous variances was chosen as the variance structure. These variables were also analyzed by subgroups for sex and hypertension. A per-protocol analysis was also performed on all participants at baseline and at the 8-week follow-up. The fixed effect models were adjusted for energy intake in sodium and potassium excretion models, and skin phenotype in blood pressure models. Mean differences were estimated with 95% CI.

## 3. Results

### 3.1. Participants

**Figure 2:**
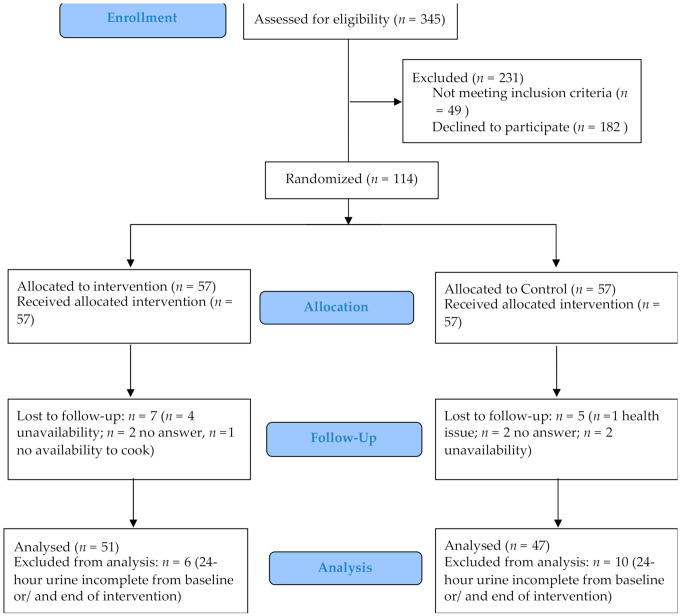
Flow diagram of participants in an intervention to reduce salt intake.

From 345 screened people, 231 were excluded for not meeting the inclusion criteria (*n* = 49) or refusing to participate in the study (*n* = 182). There were no significant differences in age, sex, education level, marital status, and whether they are hypertensive between people who refused and those who agreed to participate in the study. A total of 114 participants were included to be randomized, of which 57 were allocated to the intervention group and 57 were allocated to the control group. During the intervention, 11% of participants dropped out: seven participants in the intervention group and five in the control group. There was no significant difference between participants who were lost during the intervention or follow-up and those who completed the study regarding age, sex, marital status, education level, and whether they are hypertensive (*p* > 0.05 for all variables). The participants’ reasons for dropping out are shown in Figure 2. For the final analysis, six participants from the intervention group and 10 participants from the control group were excluded for incomplete urine at baseline and/or at the end of the intervention (Figure 2).

Data collection for the study took place during the global SARS-CoV-2 pandemic. The intervention period and the follow up period were not fulfilled during this period due to the confinements. The mean weeks in the intervention period was 14 (95 days) and the mean months in the follow-up period was 7 (206 days), with no difference between the intervention time and the follow-up time between groups.

### 3.2. Baseline Data

Table 1 shows the baseline characteristics of the participants. The mean age was 48, and 54.4% were women. Most participants were married, had higher education, were non-smokers, and on average consumed less than one alcoholic beverage per week. On average, participants had a BMI of 26 kg/m^2^, an energy intake of 2033 kcal/day, practiced 60 min per week of moderate to vigorous physical activity, and 39% were hypertensive. There were no significant differences between the intervention group and the control group (Table 1).

### 3.3. Outcomes

At baseline, only 21.1% of the participants complied with the World Health Organization sodium intake recommendations (<2000 mg/day), at the end of the intervention, 23.3% complied with the recommendations.

**Figure 3:**
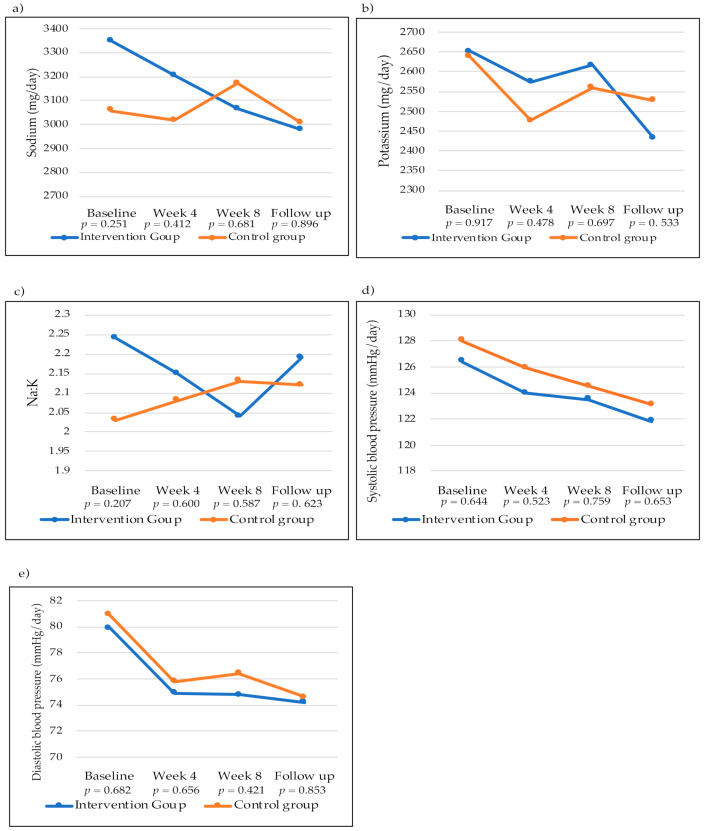
Two-dimensional (2D) line graphs with markers of the means of the four assessments of sodium and potassium excretion, Na:K ratio, and blood pressure in the intervention and control groups performed throughout the study. (**a**) Urinary sodium excretion (mg/day) of the intervention and control groups; (**b**) Urinary potassium excretion (mg/day) of the intervention and control groups; (**c**) Na:K ratio of the intervention and control groups; (**d**) Systolic blood pressure (mmHg/day) of the intervention and control groups; (**e**) Diastolic blood pressure (mmHg/day) of the intervention and control groups. *p*-Adjusted mean difference between intervention group and control group (sodium and potassium excretion and Na:K ratio were adjusted for energy intake and blood pressure was adjusted for skin phenotype).

Figure 3 demonstrates the mean excretion of sodium, potassium, Na:K ratio, and blood pressure from baseline to follow-up. Urinary sodium excretion decreased in the four assessments in the intervention group, while in the control group, it increased from baseline to the end of the intervention and then decreased in the follow-up assessment. However, there was no statistically significant mean difference at any time of the assessments between the groups (Figure 3a). Urinary potassium excretion showed a non-significant decrease (Figure 3b).

The Na:K ratio in the intervention group decreased from baseline to the end of the intervention and increased in the follow-up assessment. In the control group, the Na:K ratio increased during the intervention period and decreased slightly at follow-up. There were no statistically significant mean differences at any time between groups (Figure 3c). Systolic and diastolic blood pressure decreased in both groups over the four assessments, with no statistically significant mean difference at any time of the assessment between groups (Figure 3d,e).

Table 2 presents the results on 24 h urine measurements for sodium, potassium, Na:K ratio, and blood pressure within groups between baseline and the end of intervention.

Mean urinary sodium excretion decreased in the intervention group by around 336 mg/day (*p* = 0.088), corresponding to a 10% reduction in sodium excretion. The Na:K ratio also decreased by 0.2, but it was not statistically significant (*p* = 0.104). In the control group, mean sodium excretion increased by 50 mg/day (*p* = 0.792), and the Na:K ratio also increased by 0.1 (*p* = 0.583). Potassium excretion decreased in both groups at the end of the intervention, but the mean difference was not statistically significant (intervention group *p* = 0.710, control group *p* = 0.377). The mean difference for systolic blood pressure was significant in the control group (−3.7 (−7.4 to −0.0), *p* = 0.048) and for diastolic blood pressure, the mean difference was significant in both groups (intervention group −4.8 (−6.7 to −2.9), *p* < 0.001, control group −4.8 (−6.7 to −2.9), *p* < 0.001) (Table 2).

Table 3 shows the adjusted mean difference between the control group and the intervention group analyzed by intention to treat and per protocol. Data were similar in both analyses; there were no statistical differences in any of the outcomes analyzed.

Table 4 presents the adjusted mean difference in sodium and potassium excretion, Na:K ratio, and blood pressure from baseline to end of intervention and mean intervention effect in intention to treat by sex and by hypertension status.

In male hypertensive participants, there was a statistically significant mean difference of −1009 (−1876 to −142, *p* = 0.025) mg of sodium/day in the intervention group; in relation to the Na:K ratio, there was also a statistically significant mean difference of −0.9 (−1.5 to −0.3) in the intervention group. In systolic blood pressure, there was a mean difference in hypertensive men in the control group of −11.3 (−20.4 to −2.2), *p* = 0.018. Regarding diastolic blood pressure, there was a statistically significant mean difference in hypertensive men, hypertensive women, and non-hypertensive women in the intervention group and in the control group, corresponding to decreases in diastolic blood pressure. There was no statistically significant difference from baseline to the end of intervention between groups in any outcome (urinary excretion of sodium, potassium, Na:K ratio, and blood pressure), in intention to treat by sex and by hypertension status (Table 4).

## 4. Discussion

This intervention to reduce salt intake with an innovative dispenser (Salt Control H) that offers doses of salt according to age characteristics and the number of meals prepared appears to decrease sodium intake and improve the Na:K ratio in hypertensive men from the intervention group, although no significant differences were found for these urinary markers in relation to the control group. Nevertheless, this intervention can be considered as part of the solution to effectively reduce the consumption of salt, which remains one of the main priorities, including in clinical populations such as people with hypertension. There was no effect on potassium excretion in both groups. Diastolic blood pressure decreased in both groups.

The mean urinary sodium excretion of our sample at baseline was 3221 mg/day, which corresponds to more than 60% of the recommended intake by the World Health Organization. This high sodium intake is similar to sodium consumption reported by other authors in other countries [23,24,25].

During the intervention, particularly in some periods, a gradual reduction in sodium excretion in the intervention group and an increase in the control group, also in the Na:K ratio, was verified, however without statistical significance when comparing the groups. The results of per-protocol analysis were comparable with intention-to-treat, indicating the completeness of most urine collections. We believe that intervention with the Salt Control H equipment to reduce salt consumption was limited due to lockdown during the pandemic. The lockdown implemented during the pandemic changed people’s food preferences, and there is evidence that there has been an increase in the consumption of salt and sodium-rich foods in different countries [26]. People ingested more processed foods such as snack foods, canned goods, and instant foods, most of which were high in sodium [27]. The change in consumer behavior was driven by fears of being infected during trips to the supermarket, inflated prices for fresh produce, limited supplies, preference for foods with a long shelf life, and emotional eating [26]. Furthermore, it was reported that sodium intake not only increased during lockdown but also increased after the lockdown [28]. Together, these dietary and nutritional changes during the COVID-19 pandemic may have impacted the magnitude of urinary sodium and Na:K ratio reduction in the intervention group. For the same reason, potassium excretion decreased in both groups during the intervention period and in the follow-up assessment, and this could be due to the change in eating behavior of people in the lockdown when they decreased the intake of fresh foods such as vegetables [28], which are rich in potassium. The Na:K ratio also decreased although it was statistically significant only in hypertensive men in the intervention group. The Na:K ratio is considered a relevant measure when assessing the risk of developing high blood pressure and cardiovascular disease [29].

Systolic and diastolic blood pressure decreased in both groups over time, which may also be a consequence of lockdown as described in other studies [30,31]. In addition, a meta-analysis of randomized trials of salt reduction showed that reducing 1 g of salt per day decreased systolic blood pressure by about 1 mmHg [3].

This study can be defined as a food education intervention that combines one innovative equipment with cooking instructions and tips to reduce the salt content in food preparation that was used in clinical usual care. Previous studies have reported the effectiveness of cooking instruction in decreasing salt intake. A study of 35 housewives and 31 family members decreased 1.19 g/day of salt in the intervention group compared to the control group (*p* = 0.034) by giving cooking classes focusing on the amount of salt in a meal and instructions on how to cook with less salt [32]. Another study of 753 participants at high risk for cardiovascular disease reduced 0.66 g/day of salt between groups (*p* = 0.03) with an education program that included cooking instructions using a pocket-sized digital salt meter to display the content of their food daily. In addition, systolic blood pressure also decreased by 7.55 mm Hg, *p* < 0.01 [33]. In another study of 403 participants, the intervention group received an improvement salt-restriction-spoon and health education and reduced daily salt intake by 1.42 g [34].

Meeting the World Health Organization’s recommendations for salt intake (<5 g/day) remains a problem, and the main sources of salt intake continue to be varied, including bread products, cereals and grains, meat products, and dairy products, but the discretionary use of salt has marked variation across the world and is a major source of dietary salt in many countries, including Portugal [9]. The use of equipment that offers doses of salt according to the number of persons and their age group could be considered as a promising strategy to be part of the solution for reducing salt population intake, considering that it responds to people’s difficulty in measuring and knowing the maximum amount of salt that is recommended for cooking their meals, being especially useful to tackle added salt.

Our results have important implications for public health. Salt Control H, in addition to providing salt doses according to the daily salt recommendations, is an educational tool that can be used by health professionals to teach people about the maximum dose of salt they can use to prepare their meals. It is important to emphasize that in order to achieve the greatest reduction in salt consumption by the population, it is important to combine with other strategies, namely working with the food industry to reduce the amount of salt added to processed foods.

A strength of the study was that sodium consumption was assessed through 24 h urinary excretion, and multiple measurements were performed during intervention period. It is the gold standard method as approximately 90 to 95% of ingested sodium is excreted in the urine [35]. Although it may have inaccuracies and collection errors [36], it is the most reliable method [37]. Another strength is that the study design followed the recommendations and best practices of clinical trials.

Our study has limitations. First, as already mentioned, data collection was carried out during the period of the COVID-19 pandemic, changing the duration of intervention and follow-up, and we were not able to reach the calculated sample size. Second, participation in the study was limited to employees of a university, most had a high level of education, and the results are not generalizable to the general population. Third, although our effort was that the participants in the control group did not change their behavior, we must admit that changing the intake is always possible just by being a participant in a trial.

## 5. Conclusions

Our study showed that the tested equipment that provides salt doses according to the number of persons and their age, accompanied by general written cooking instructions, decreased sodium and improve the Na:K urinary excretion in hypertensive men from the intervention group, although no significant differences were found for these urinary markers in relation to the control group. It will be important in the future to test this intervention with a larger sample size and in a period after the pandemic.

## Figures and Tables

**Table 1:** Sociodemographic, lifestyle, clinical, anthropometric, and nutritional characteristics of 114 adults participating in a randomized control trial.

Participants’ Characteristics	Total (*n* = 114)	Intervention Group (*n* = 57)	Control Group (*n* = 57)	*p*-Value
Age (mean ± SD)	48 ± 11	47 ± 10	49 ± 11	0.178
Women (%)	54.4	52.6	56.1	0.707
Education (%)				0.166
No higher education	13.2	8.8	17.5	
Higher education	86.8	91.2	82.5	
Marital Status (%)				
Single	16.7	17.5	15.8	0.813
Married	68.4	64.9	71.9	
Divorced	11.4	14	8.8	
Widow/er	3.5	3.5	3.5	
Hypertensive (%)	38.6	42.1	35.1	0.442
Body mass index, kg/m^2^ (mean ± SD)	26.0 ± 3.9	25.8 ± 4.0	26.2 ± 3.9	0.587
Energy intake (kcal) (median [P25; P75])	2033 [1632; 2596]	2042 [1697; 2629]	2007 [1559; 2461]	0.375
Moderate and/or vigorous physical activity/week (minutes per week) (median [P25; P75])	60 [0; 240]	70 [0; 255]	60 [0; 210]	0.804
Skin phenotypes ^1^ (%)				
Type 1	1.8	1.8	1.8	NA
Type 2	10.5	10.5	10.5	
Type 3	51.8	50.9	52.6	
Type 4	34.2	35.1	33.3	
Type 5	0.0	0.0	0.0	
Type 6	1.8	1.8	1.8	
Smoking habits (%)				
Non smoking	63.2	63.2	63.2	0.424
Smoker	12.3	15.8	8.8	
Former smoker	24.6	21.1	28.1	
Alcohol intake, drinks/week (median [P25; P75])	0.29 [0.15; 0.75]	0.29 [0.1; 0.7]	0.29 [0.14; 0.86]	0.590

^1^ Type 1—redhead with freckles; Type 2—blonde; Type 3—brunette; Type 4—Latino; Type 5—Arab; Type 6—Asian or black. SD—Standard deviation; NA—Not applicable.

**Table 2:** Adjusted mean difference in urinary excretion of sodium and potassium, Na:K ratio, blood pressure based on intention-to-treat analysis in an intervention to reduce salt intake.

Outcomes	Intervention Group				Control Group			
	Baseline (Mean [95%CI])	End of Intervention, Week 8 (Mean [95%CI])	Change from Baseline [95%CI]	*p*-Value	Baseline (Mean [95%CI])	End of Intervention, Week 8 (Mean [95%CI])	Change from Baseline [95%CI]	*p*-Value
Sodium (mg) *	3369 [3021 to 3717]	3033 [2653, 3413]	−336 [−723 to 51]	0.088	3135 [2782 to 3488]	3185 [2812 to 3558]	50 [−327 to 428]	0.792
Potassium (mg) *	2658 [2454 to 2862]	2615 [2403, 2828]	−43 [−270 to 184]	0.710	2665 [2457 to 2871]	2565 [2357 to 2773]	−99 [−321 to 123]	0.377
Na:K *	2.2 [2.0 to 2.5]	2.0 [1.8 to 2.2]	−0.2 [−0.5 to 0.0]	0.104	2.1 [1.8 to 2.3]	2.1 [1.9 to 2.4]	0.1 [−0.2 to 0.4]	0.583
SBP (mmHg) **	126.2 [121.4 to 131.0]	123.0 [118.8 to 127.2]	−3.2 [−7.0 to 0.6]	0.094	127.7 [122.9 to 132.6]	124.1 [119.9 to 128.2]	−3.7 [−7.4 to −0.0]	0.048
DBP (mmHg) **	79.7 [76.5 to 82.8]	74.9 [72.0 to 77.7]	−4.8 [−6.7 to −2.9]	<0.001	81.2 [78.0 to 84.4]	76.4 [73.6 to 79.2]	−4.8 [−6.7 to −2.9]	<0.001

* Adjusted for energy intake. ** Adjusted for skin phenotype. SBP—systolic blood pressure; DBP—diastolic blood pressure; CI—confidence interval. *p*-value calculated using linear mixed models.

**Table 3:** Mean intervention effect (adjusted mean difference (intervention vs. control)) in intention-to-treat and per-protocol analysis.

Outcomes	Intention to Treat		Per Protocol	
	Adjusted Difference [95%CI] (Intervention vs. Control)	*p*-Value	Adjusted Difference [95%CI] (Intervention vs. Control)	*p*-Value
Sodium (mg) *	−152 [−684 to 380]	0.350	−163 [−753 to 428]	0.585
Potassium (mg) *	50 [−247 to 347]	0.738	−58 [−223 to 107]	0.487
Na:K *	−0.1 [−0.4 to 0.2]	0.426	−0.2 [−0.5 to 0.2]	0.267
SBP (mmHg) **	−1.0 [−6.9 to 4.8]	0.729	−1.2 [−7.7 to 5.3]	0.715
DBP (mmHg) **	−1.6 [−5.6 to 2,4]	0.441	−1.7 [−6.1 to 2.7]	0.445

* Adjusted for energy intake. ** Adjusted for skin phenotype, Fitzgerald scale. SBP—systolic blood pressure; DBP—diastolic blood pressure; CI—confidence interval. *p*-value calculated using linear mixed models.

**Table 4:** Adjusted mean difference in sodium and potassium excretion, Na:K ratio, and blood pressure from baseline to end of intervention and mean intervention effect (adjusted mean difference (intervention vs. control)) in intention to treat by sex and by hypertension status.

			Intervention Group		Control Group			
Outcomes	Sex	Hypertension	Adjusted Difference [95%CI] (Change from Baseline)	*p*-Value	Adjusted Difference [95%CI] (Change from Baseline)	*p*-Value	Adjusted Difference [95%CI] (Intervention vs. Control)	*p*-Value
Sodium (mg) *	Women	Yes	105 [−795 to 1013]	0.810	109 [−795 to 1013]	0.798	−536 [−1916 to 845]	0.420
		No	−253 [−761 to 256]	0.320	211 [−263 to 685]	0.320	42 [−638 to 721]	0.902
	Men	Yes	−1009 [−1876 to −142]	0.025	26 [−977 to 1030]	0.957	−649 [−1778 to 481]	0.245
		No	127 [−808 to 1062]	0.781	−126 [−977 to 726]	0.764	175 [−1036 to 1385]	0.768
Potassium (mg) *	Women	Yes	−31 [−543 to 482]	0.899	−157 [−674 to 359]	0.518	−194 [−820 to 433]	0.518
		No	126 [−255 to 507]	0.506	113 [−247 to 473]	0.528	135 [−285 to 555]	0.518
	Men	Yes	199 [−348 to 745]	0.453	−316 [−933 to 301]	0.296	−24 [−894 to 847]	0.955
		No	−474 [−958 to 11]	0.055	−154 [−594 to 285]	0.473	136 [−566 to 839]	0.692
Na:K *	Women	Yes	0.2 [−0.4 to 0.9]	0.473	0.3 [−0.4 to 0.9]	0.369	−0.3 [−1.3 to 0.7]	0.543
		No	−0.3 [−0.7 to 0.017]	0.062	0.0 [−0.3 to 0.4]	0.811	−0.1 [−0.5 to 0.3]	0.562
	Men	Yes	−0.9 [−1.5 to −0.3]	0.007	0.3 [−0.4 to 1.1]	0.376	−0.4 [−1.2 to 0.4]	0.292
		No	0.4 [−0.3 to 1.1]	0.442	−0.2 [−0.3 to 1.1]	0.203	0.2 [−0.4 to 0.8]	0.512
SBP (mmHg) **	Women	Yes	−10.5 [−23.7 to 2.6]	0.109	−7.3 [−20.3 to 5.6]	0.109	−9.3 [−27.4 to 8.7]	0.287
		No	−3.4 [−8.6 to 1.9]	0.203	1.6 [−3.2 to 6.6]	0.501	−3.7 [−10.5 to 3.2]	0.284
	Men	Yes	−6.7 [−14.8 to 1.4]	0.100	−11.3 [−20.4 to −2.2]	0.018	0.8 [−11.9 to 13.5]	0.901
		No	5.2 [−1.1 to 11.4]	0.101	−2.9 [−8.7 to 2.9]	0.306	2.5 [−8.4 to 13.4]	0.642
DBP (mmHg) **	Women	Yes	−8.4 [−15.1 to −1.6]	0.018	−8.4 [−15.1 to −1.6]	0.018	−0.6 [−8.9 to 7.6]	0.872
		No	−2.6 [−5.0 to −0.1]	0.040	−2.6 [−5.0 to −0.1]	0.040	−0.7 [−5.2 to 3.8]	0.758
	Men	Yes	−8.1 [−12.6 to −3.6]	0.001	−8.1 [−12.6 to −3.6]	0.001	−5.9 [−12.9 to 1.0]	0.089
		No	−2.6 [−5.6 to 0.5]	0.097	−2.6 [−5.6 to 0.5]	0.097	−0.7 [−9.4 to 7.9]	0.866

* Adjusted for energy intake. ** Adjusted for skin phenotype, Fitzgerald scale. SBP—systolic blood pressure; DBP—diastolic blood pressure; CI—confidence interval. *p*-value calculated using linear mixed models with an intention-to-treat approach.

## Data Availability

The data presented in this study are available on request from the corresponding author. The data are not publicly available due to ethical.
